# Particle Deposition in a Child Respiratory Tract Model: *In Vivo* Regional Deposition of Fine and Ultrafine Aerosols in Baboons

**DOI:** 10.1371/journal.pone.0095456

**Published:** 2014-04-30

**Authors:** Iolanda Albuquerque-Silva, Laurent Vecellio, Marc Durand, John Avet, Déborah Le Pennec, Michèle de Monte, Jérôme Montharu, Patrice Diot, Michèle Cottier, Francis Dubois, Jérémie Pourchez

**Affiliations:** 1 LINA, EA 4624, Saint-Etienne, France; 2 Ecole Nationale Supérieure des Mines, CIS-EMSE, LINA EA 4624, Saint-Etienne, France; 3 SFR IFRESIS, Saint-Etienne, France; 4 EA6305, CEPR, Faculté de médecine, Université François Rabelais, Tours, France; 5 DTF-Aerodrug, Faculté de médecine, Tours, France; 6 Centre Hospitalier Emile Roux, Le Puy en Velay, France; 7 Université Jean Monnet, Faculté de Médecine, Saint-Etienne, France; 8 CHU de Saint-Etienne, Saint-Etienne, France; 9 Université de Lyon, Saint-Etienne, France; 10 Service de Pneumologie, CHRU de Tours, Tours, France; University of Calgary & ProvLab Alberta, Canada

## Abstract

To relate exposure to adverse health effects, it is necessary to know where particles in the submicron range deposit in the respiratory tract. The possibly higher vulnerability of children requires specific inhalation studies. However, radio-aerosol deposition experiments involving children are rare because of ethical restrictions related to radiation exposure. Thus, an *in vivo* study was conducted using three baboons as a child respiratory tract model to assess regional deposition patterns (thoracic region vs. extrathoracic region) of radioactive polydisperse aerosols ([d16–d84], equal to [0.15 µm–0.5 µm], [0.25 µm–1 µm], or [1 µm–9 µm]). Results clearly demonstrated that aerosol deposition within the thoracic region and the extrathoraic region varied substantially according to particle size. High deposition in the extrathoracic region was observed for the [1 µm–9 µm] aerosol (72%±17%). The [0.15 µm–0.5 µm] aerosol was associated almost exclusively with thoracic region deposition (84%±4%). Airborne particles in the range of [0.25 µm–1 µm] showed an intermediate deposition pattern, with 49%±8% in the extrathoracic region and 51%±8% in the thoracic region. Finally, comparison of baboon and human inhalation experiments for the [1 µm–9 µm] aerosol showed similar regional deposition, leading to the conclusion that regional deposition is species-independent for this airborne particle sizes.

## Introduction

The likelihood of the presence of primary nano-objects, *i.e.*, manufactured structures such as nanofibers, nanoplates, and nanoparticles [1], in the air seems to be low. There are indications that the lifetime of primary airborne nanoparticles (considered to be <100 nm) is limited by a rapid coagulation process. Larger airborne aggregates (or agglomerates) can be formed. Some authors have suggested the attachment of the nanoparticles to larger background particles and the mutual coagulation of the nano-sized aerosols [2], [3]. Concentration dependency was also observed: the higher the concentrations, the more rapid the coagulation. In real-world workplace conditions, the lifetime of primary airborne nanoparticles is limited, and it would be more likely for individuals to be exposed to nanoparticle agglomerate aerosol. Thus, some authors have suggested that occupational exposure to the release of nano-size particles in the air might be mimicked by a submicronic aerosol with two populations, one of approximately 200 to 600 nm and one of approximately 2000 to 3000 nm, which might imply that workers inhale aerosol submicron-size fractions rather than aerosol nano-size fractions [4].

To relate exposure to adverse health effects, it is crucial to assess the deposition pattern of inhaled particles in the respiratory tract. The identification of deposition sites is a major determinant of particle biopersistence, clearance from the respiratory tract, the dose pattern in tissues, and the resultant biological effects. Spaces like the extrathoracic airways show fast mucociliary clearance, with residence time in the range of hours to 1 day. Thus, the deposition of submicron-size nanoparticle agglomerates in this region may have less toxicological impact compared to alveolar deposition, with long clearance times in the range of days to months for poorly soluble particles. However, high alveolar deposition enables possible systemic translocation of primary nanoparticles [5].

As a result, it would be relevant for risk assessment purposes to know the size of submicron-size nanoparticles agglomerates in the 200-nm to 600-nm range that are preferentially deposited in the pulmonary alveolar spaces. The region of the respiratory tract affected by particle deposition largely depends on particle properties (*e.g.*, micrometer-size vs. submicron-size particles), and also on anatomic and physiologic properties (*e.g.*, adult vs. child respiratory tract model) [5], [6]. Despite behavioral and physiological differences between adults and children, experimental investigations of the regional deposition pattern of inhaled particles within the respiratory tract have mainly focused on adults, with children typically addressed by mathematical modeling. Application of models of inhaled particle deposition is largely based on extrapolating anatomical and physiological data from young adults to match the changes observed during growth and aging [7]. For micrometer-size aerosol, the particle deposition models consistently predict greater tracheobronchial aerosol deposition and lesser pulmonary deposition for children than that calculated for adults [8]. Only approximately 50 studies that measure the respiratory tract deposition of particles smaller than 300 nm have been reported [9]. However, with age, there may be significant variations in species-specific particle deposition factors [10], [11]. Thus, children may receive a greater internal dose of submicron nanoparticle agglomerates than do adults because of different airway architecture and greater ventilation rate per body weight or lung surface area [12]. Moreover, metabolic differences may result in different tissue burdens. This can lead to differences between children and adults regarding submicron-size nanoparticle agglomerate doses, elimination, and toxicity.

This possibly higher vulnerability of children requires designing specific inhalation studies. In this context, baboons are generally considered to be the best animal model for extrapolating data regarding aerosol deposition in human airways [13], [14]. A morphometric comparison of baboon airways with the respiratory tract geometry of a 2-year-old child suggests a functional interspecific relationship between the nasal structure, cross-sectional area, and tracheobronchial region [15], [16]. The main objective of this article was to assess doses of inhaled submicron-size aerosol in baboons (*i.e.*, an animal model of the human child respiratory tract). Therefore, an *in vivo* study was conducted using three baboons to assess regional deposition patterns (thoracic [TH] region vs. extrathoracic [ET] region) of radioactive polydisperse aerosols ([d16–d84] equal to [0.15 µm–0.5 µm], [0.25 µm–1 µm], or [1 µm–9 µm]), which are quite well-representative of nanoparticle agglomerates observed in the case of airborne nanoparticle exposure [4].

## Materials and Methods

### Aerosol Generation and Characterization

The following commercial jet medical nebulizers were used to generate micrometric and submicrometric aerosols: Atomisor NL11 (DTF Medical, Saint-Etienne, France); modified Sidestream (Ref 12NEB400; Philips Respironics, Tangmere, England); and a Nanoneb (DTF Medical). Nebulizers were loaded with 2 mL diethylenetriamine pentaacetic acid (DTPA) solution (Pentacis; CIS Bio International, Gif-sur-yvette, France) containing 74 MBq technetium 99m (^99m^Tc). Aerosols were generated by using an air tank (Air Liquide, Paris, France) with a flow rate of 8 L/min.

Particle size distributions of the analyzed radioactive aerosols were determined by using a gamma camera (Ecam; Siemens) coupled to a specific electrical low-pressure impactor (ELPI; Dekati, Kangasala, Finland). The ELPI was specifically devoted to airborne nanoparticles. With this device, operated at an air flow rate of 10 L/min, particles are impacted depending on their inertia-related aerodynamic diameter at one of the 12 size fraction stages of the impactor (range, 10 µm–7 nm). To operate the ELPI at the specific flow rate, 2 L/min of dilution air (at controlled relative humidity) was added to the nebulizer flow rate. Before each measurement, the 12 ELPI impaction stages were cleared. The corona charger was turned off during experiments to avoid the artifact effect of particles charging and electrical detection. Consequently, the particle distribution was characterized using the ELPI with the particles not being electrically charged. During aerosol measurements, each nebulizer was directly connected with the ELPI impactor system. The aerosol produced was then collected and sampled because the aerosolized particles are impacted at different stages according to their inertia related to their aerodynamic diameter. At the end of nebulization, defined as 1 minute after the beginning of the sputtering, the radioactivity from aerosol particles deposited in each size-specific stage was quantified by scintigraphy imaging. The ELPI directly measured the activity in the particle size distribution once the activity distribution with a median size was able to be described. Thus, the ELPI device allowed the measurement of the activity median aerodynamic diameter (AMAD); however, at the same time, it also permitted quantifying the total amount of radioactivity delivered by the nebulizer to calculate the emitted nebulizer fraction sum of the activity during the 12 stages and the human throat model inserted before the ELPI.

In short, the emitted nebulizer fraction, the particle size distribution, the AMAD with geometric standard deviation (GSD), the fine particle (FP) fraction (including all particles with an aerodynamic diameter <2.5 µm), the ultrafine particle fraction (including all particles with an aerodynamic diameter <0.1 µm), and the [d16–d84] particle size range (where d16 and d84 are the particle diameters at the 16% and 84% size cut-offs of the cumulative distribution, respectively) were calculated from radioactivity deposited at each ELPI stage. Furthermore, raw current charges of airborne particles were also analyzed. Although all studied aerosols were exclusively composed of positively charged particles, the [1 µm–9 µm] aerosol presented higher raw current values.

The ELPI measurement neglects any hygroscopic changes of the nebulizer droplets. However, the particle sizes measured by the ELPI are subject to possible size changes.

### Animals and Housing Conditions

*In vivo* experiments were performed with three healthy baboons (*Papio anubis*) weighing 10 to 14 kg (age, 6.3±0.5 years). Animals were obtained from an official supplier (Station de primatologie de Rousset, Marseille, France). Animal accommodations were in accordance with the last European legislation (Directive 2010/63/UE). Baboons were housed as a social group in three stainless-steel mesh cages (2×2×2 m) that were maintained with the doors open. Temperature (22°C±2°C) and relative humidity (range, 30%–70%) of the room were measured, controlled, and recorded daily. An artificial lighting cycle of 12 hours (from 8:00 am to 8:00 pm) was maintained. Each day, the animals were offered an expanded commercial primate diet (808000; SDS, St. Gratien, France).

No animals were killed during this study. Enrichment is widely believed to improve the psychological health of nonhuman primates. Attention to the psychological well-being of laboratory primates derives from ethical concerns for the welfare of creatures and from the need to ensure a healthy subject for research. Baboons were kept in a harmonious group with music playing from speakers and wildlife films shown on television (once per week) to allow social and environmental enrichment. Moreover, to address their arboreal nature, we provided laboratory baboons with access to a place to sit or climb above floor level. Their diets were also supplemented with fresh fruits. Their main drinking water was available *ad libitum* from automatic dispensers. Baboons have hands that resemble those of humans, and they can spend many minutes at a time manipulating their environment. To provide opportunities for manipulation, we provided baboons with cage toys that were regularly changed.

### *In Vivo* Experimental Design and Image Acquisition

One of the main challenges for calibration and comparison of instruments for lung deposition experiments is the lack of a realistic common “standard” or “reference” method. A recent review of the measurement techniques for respiratory tract deposition of airborne nanoparticles recommended experimental procedures to minimize errors [9]. This study takes into account some of these suggestions to limit biases attributable to measurement problems. The experimental protocol was conducted according to National Institutes of Health *Guidelines for the Care and Use of Laboratory Animals* and according to the approval of a local ethics board (protocol was recorded by the French National Committee of Ethical Reflection on Animal Experiments). Baboons were placed on a special chair under anesthesia via intramuscular injection of xylazine (1 mg/kg) and ketamine (5 mg/kg). The animals were sedated, but they were kept awake while aerosol was administered through a tight-fitting face mask (Ref 93815028; Temsega, France) specifically designed for baboons with a combination of oral and nasal inhalation routes. Each baboon inhaled an aerosol twice for each of the three different types of nebulizers selected for this study, resulting in six inhalations per nebulizer. The order of the nebulizers tested was randomized for each baboon. The nebulizer charge was controlled by counting the radioactivity in the syringe using a gamma counter (Capintec; France) before and after charging. The nebulizer was then connected to the face mask and to an expiratory filter to avoid air contamination. It was operated until 1 minute after the aerosol began to sputter. Immediately after aerosol delivery, the animals and the circuit components were scanned using a gamma camera (Ecam; Siemens). A 120-second posterior static view was acquired on a 128×128 matrix. The amount of ^99m^Tc-DTPA deposited in the lungs was determined from the digitized images. This *in vivo* study was conducted under the approval of the ethics board at the Medicine University of Tours in France (file number 2010/20). Baboon tidal volume was measured with a pneumotach (Dyn R, Muret, France) in the three studied baboons (Table 1). Deposition in the ET and TH regions was expressed as a fraction of the inhaled aerosol amount, which is more relevant to ambient aerosol exposure estimation than using emitted nebulizer fraction. The inhaled aerosol fraction was estimated by dividing baboon minute ventilation (1.8 L/min) by nebulizer flow rate (8 L/min), neglecting the inhalability losses during the experiments.

**Table 1:** Breathing parameters of the three studied baboons (mean ± standard deviation).

Breathing rate (breaths/min)	35±7
Tidal volume (mL)	54±9
Inspiratory time to expiratory time (I:E)	0.41±0.09: 0.59±0.09
Inspired air (L/min)	1.8±0.15
Age (years)	6.3±0.5
Weight (kg)	12.3±1.2

### Image Analysis

The strategy to assess regional deposited amounts of aerosol in the ET and the TH regions is a first step to differentiate the respiratory tract into different regions. The scintigraphic images of aerosol deposition in baboons were post-treated with a 9-point convolution mask filter to reduce background noise without loss of image information. The regions of interest were determined manually, delimiting two main regions, the TH region and the ET region [17]. Background noise was subtracted from measured counts of radioactivity within regions of interest. All images presented a good count rate, giving the data a relative accuracy of 3%. Furthermore, corrections for physical decay of ^99m^Tc were made for all measurements. Tissue attenuation coefficients were also taken into account. Lung tissue attenuation correction factors were determined from lung perfusion imaging of each baboon using pertechnetate-macroaggregated albumin. Stomach tissue attenuation correction factors were determined from administration of 5 mL ^99m^Tc-DTPA via a catheter directly into the stomach of each baboon. Head tissue attenuation correction factors were determined by placing a tube containing 5 mL ^99m^Tc-DTPA in the mouth of each baboon. All images were recorded from the anterior view of the baboon.

### Statistical Analysis

Statistics were performed using GraphPad Prism Software version 5 (GraphPad,). A Bonferroni multiple comparison test was used to compare the data generated (*p*<0.05 was considered statistically significant).

### Ethics Approval

This study was approved by the local ethics board of Tours University (file number 2010/20) and was recorded by the French National Committee of Ethical Reflection on Animal Experiments (CNREEA, under number CEEAVdL-19). The ARRIVE (Animal Research: Reporting *In Vivo* Experiments) guidelines are available in Checklist S1 to improve the reporting of animal experiments.

## Results

### Particle Size Distribution

**Figure 1:**
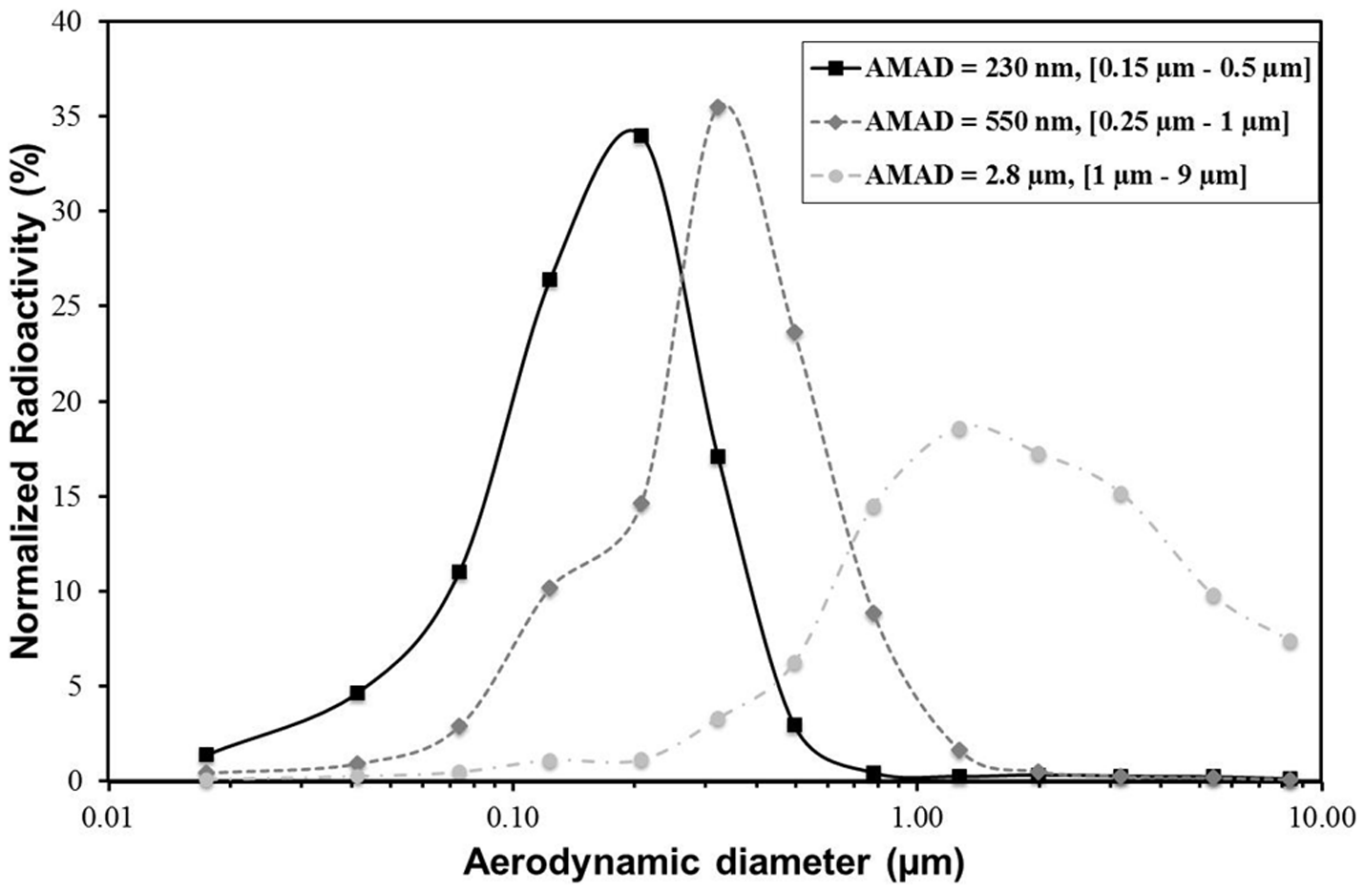
Activity size distributions of the studied aerosols measured using gamma camera detection coupled to the cascade electrical low-pressure impactor. AMAD, activity median aerodynamic diameter.

Particle size distributions of the three polydisperse aerosols are shown in Figure 1 and summarized in Table 2. Because the particle size distributions are broad and quite far from a monodisperse distribution, we chose to characterize the aerosol distribution by the means of the [d16–d84] particle size range. AMAD of 2.8 µm (GSD of 3.2; [d16–d84]  =  [1 µm–9 µm]; Atomisor NL11), 550 nm (GSD of 2.1; [d16–d84] = [0.25 µm–1 µm]; modified Sidestream), and 230 nm (GSD of 1.6; [d16–d84] = [0.15 µm–0.5 µm]; Nanoneb) were obtained. Almost all particles produced by the [0.15 µm–0.5 µm] aerosol (98.6%) and the [0.25 µm–1 µm] aerosol (98.3%) were smaller than 2.5 µm, which corresponded to the FP that are likely to be deposited in the lungs [6], [18]. In contrast, less than half of the [1 µm–9 µm] aerosol (47%) was considered FP. All things considered, the [0.15 µm–0.5 µm] aerosol produced the larger amount of ultrafine particle fractions smaller than 0.1 µm (7.3%), almost four-times higher than the [0.25 µm–1 µm] aerosol and 20-times higher than the [1 µm–9 µm] aerosol (Table 2).

**Table 2:** Features of the aerosols inhaled by the baboons to assess the impact of particle size on the *in vivo* regional distribution.

Nebulizers	AMAD (GSD) ELPI and gamma camera	Particles <2.5 µm (FP)	Particles <1 µm	Particles <0.5 µm	Particles <0.1 µm (UFP)	[d16–d84]
NL11	2.80 µm (3.2)	47.0%±4.3%	13.6%±1.6%	4.7%±0.6%	0.4%±0.05%	[1 µm–9 µm]
Modified Sidestream	550 nm (2.1)	98.6%±0.2%	89.2%±2%	47.9%±4.4%	1.7%±0.2%	[0.25 µm–1 µm]
Nanoneb	230 nm (1.6)	98.3%±0.4%	97.5%±0.5%	86.6%±1.8%	7.3%±1.2%	[0.15 µm–0.5 µm]

AMAD, activity median aerodynamic diameter; ELPI, electrical low-pressure impactor; FP, fine particle; GSD, geometric standard deviation; UFP, ultrafine particle.

### *In Vivo* Regional Deposition: ET/TH Deposition Balance

**Figure 2:**
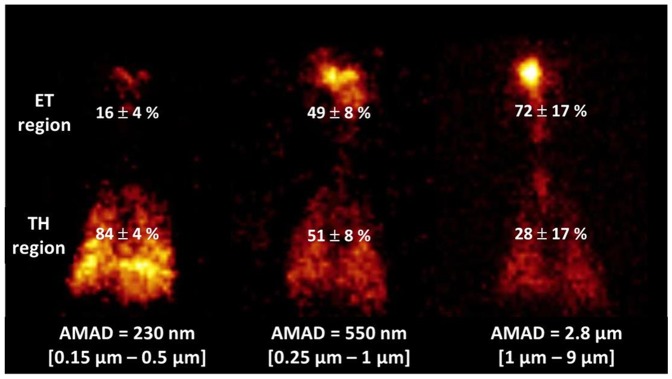
*In vivo* inhalation experiments using baboons. Representative scintigraphic images obtained for the three studied aerosols. All images are from the same baboon. Total aerosol depositions (%) for the extrathoracic (ET) and thoracic (TH) regions are indicated. Activity median aerodynamic diameter (AMAD) and [d16, d84] were noticed for each aerosol generated.

In the submicron-size range studied, results demonstrated that the TH region deposition decreased as particle sizes increased (Figure 2). A huge deposition in the ET region was found for the [1 µm–9 µm] aerosol (72%±17% of the total aerosol fraction deposited), whereas the [0.15 µm–0.5 µm] aerosol showed the smallest ET region deposition (only 16%±4% of the total aerosol fraction deposited). As a result, aerosols with smaller AMAD (*i.e.*, 230 nm or [0.15 µm–0.5 µm]) were associated almost exclusively with thoracic deposition (84%±4% of the total aerosol fraction deposited). Finally, [0.25 µm–1 µm] aerosol with AMAD of 550 nm showed intermediate behavior, with 49%±8% of the total aerosol deposition in the ET region and 51%±8% in the TH region. Statistical analysis (summarized in Table 3) confirmed that particle size is the main factor influencing aerosol deposition in the ET and TH regions. Results exhibited three different patterns of aerosol deposition (*i.e.*, three different ET/TH deposition balances) for the particle sizes studied (*i.e.*, 2.8 µm, 550 nm, and 230 nm).

**Table 3:** Statistical analysis by Bonferroni multiple comparison test: Impact of the airborne particle size (*i.e.*, [d16–d84]) on the experimental ET and TH regional depositions.

	[1 µm–9 µm] versus [0.25 µm–1 µm]	[1 µm–9 µm] versus [0.15 µm–0.5 µm]	[0.25 µm–1 µm] versus [0.15 µm–0.5 µm]
ET deposition	*p*<0.001	*p*<0.001	*p*<0.001
TH deposition	*p*<0.001	*p*<0.001	*p*<0.001

ET, extrathoracic; TH, thoracic.

### *In Vivo* Regional Distribution: Aerosol Deposited and Aerosol Exposure

**Figure 3:**
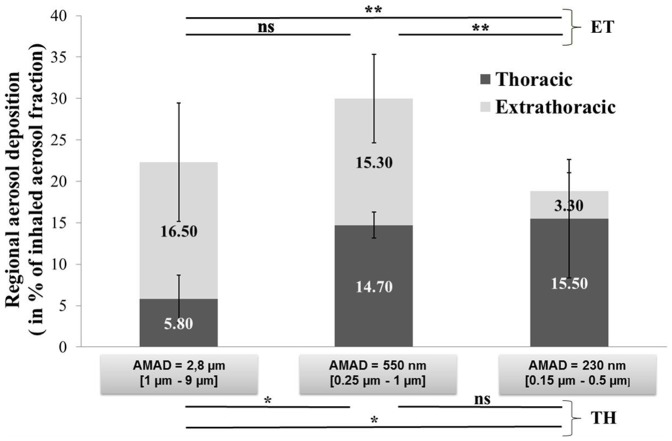
*In vivo* results of aerosol deposition within the extrathoracic (ET) and thoracic (TH) regions in terms of the inhaled aerosol fraction (*p<0.05, **p<0.01, and ***p<0.001 by Bonferroni multiple comparison test). AMAD, activity median aerodynamic diameter; NS, not significant.

The ET/TH deposition balance led to interesting data and was also useful for determining the mass of aerosol deposited as a function of the inhaled aerosol fraction (*i.e.*, the amount of particles inhaled by the baboons) rather than the emitted aerosol fraction (*i.e.*, the amount of particles delivered by the nebulizer in the atmosphere during aerosol exposure) (Table 4). For the [1 µm–9 µm] aerosol, we observed a five-fold higher emitted fraction (44.5%±1.5% vs. 9.3%±3%) compared with the [0.25 µm–1 µm] aerosol and a 10-fold higher emitted fraction compared with the [0.15 µm–0.5 µm] aerosol (44.5%±1.5% vs. 4.2%±1%). Thus, results for the deposited fractions can be expressed in terms of the percentage of the inhaled aerosol fraction (Figure 3). The results show a great impact of the particle size on the relative deposition efficiency. In fact, a higher total deposition fraction was obtained for the [0.25 µm–1 µm] aerosol, with 1.6-fold and 1.35-fold higher total deposition fractions than the [0.15 µm–0.5 µm] and [1 µm–9 µm] aerosols, respectively. We demonstrated that the fraction deposited in the ET region was statistically constant for [1 µm–9 µm] or [0.25 µm–1 µm] aerosols (16.5%±7.1% and 15.3%±5.3%, respectively), whereas it significantly decreased for the [0.15 µm–0.5 µm] aerosol (3.3%±2.2%). As a result, the higher regional deposition fraction in the ET region was observed for the [1 µm–9 µm] and [0.25 µm–1 µm] aerosols, with five-fold higher deposition than the [0.15 µm–0.5 µm] aerosol. Moreover, the fraction deposited in the TH region was relatively low for the [1 µm–9 µm] particles (5.8%±2.9%), but it significantly increased for the [0.25 µm–1 µm] and [0.15 µm–0.5 µm] aerosols (14.7%±1.6% and 15.5%±7.1%, respectively). Therefore, the higher regional deposition fraction in the TH region was observed for the [0.15 µm–0.5 µm] and [0.25 µm–1 µm] aerosols, with a 2.5-fold higher deposition than the [1 µm–9 µm] aerosol.

**Table 4:** Impact of airborne particle size on the emitted fraction by each nebulizer and the deposition pattern in terms of emitted aerosol fraction deposited in the ET and TH regions

Nebulizer	AMAD (GSD)	[d16–d84]	Emitted fraction (%)	TH deposition (% of emitted aerosol fraction)	ET deposition (% of emitted aerosol fraction)
NL11	2.80 µm (3.2)	[1 µm–9 µm]	44.5±1.5	1.3±0.65	3.7±1.6
Modified Sidestream	550 nm (2.1)	[0.25 µm–1 µm]	9.3±3	3.3±0.35	3.45±1.2
Nanoneb	230 nm (1.6)	[0.15 µm–0.5 µm]	4.2±1	3.5±1.6	0.75±0.5

AMAD, activity median aerodynamic diameter; ET, extrathoracic; GSD, geometric standard deviation; TH, thoracic.

## Discussion

### Impact of Airborne Particle Size on the ET/TH Deposition Balance in Terms of the Total Aerosol Fraction Deposited

Patterns of regional deposition of particles within airways remain a key experimental issue to successfully predict where pathological changes may occur or to estimate the possibility of translocation through the air–blood barrier. Numerous regional deposition data for humans were developed as a function of particle size as early as the 1960s, *e.g.*, radiolabeled methacholine studies [19], [20] and the numerous bolus studies examining regional deposition. However, many aerosol deposition experiments in humans and in laboratory animals have focused on the total deposited fraction [18], [21]–[25]. This fraction can be measured by comparing particle concentrations in the inhaled and exhaled air, but regional involvement cannot be distinguished. Additionally, studies are generally devoted to the deposition pattern within adult respiratory tract models and focus on micrometer-size particle ranges. *In vivo* studies of the regional deposition of submicron-size particles in the respiratory tract are still scarce, and aerosol deposition studies involving infants and children are even more rare, with only a few experimental studies related to children relayed in the literature [26], [27]. However, deposition is generally assessed by using inhalation of radiolabeled aerosols and gamma scintigraphy imaging, and this radiation exposure places ethical restrictions on performing such studies in children.

In this context, one of the main results obtained is the demonstration that regional aerosol deposition in a child respiratory tract model varies substantially according to the polydisperse aerosol features in the submicrometer-size particle range (Figure 2). We showed that the smallest AMAD aerosol (*i.e.*, 230 nm or [0.15 µm–0.5 µm]) led to targeted deposition in the TH region. The high proportion of small particles contained in the [0.15 µm–0.5 µm] airborne particles (86.6%±1.8% of particles <0.5 µm) (Table 2) may explain the targeted thoracic deposition and the low intersubject variability observed (Figure 2). The fraction of particles smaller than 0.5 µm appeared to be correlated with aerosol deposition in the TH region. The [0.15 µm–0.5 µm] aerosol showed a 1.8-fold higher amount of particles smaller than 0.5 µm compared with the [0.25 µm–1 µm] aerosol (86.6%±1.8% vs. 47.9%±4.4%) (Table 2) and a 1.8-fold higher deposition in the TH region (84%±4% vs. 51%±8%) (Figure 2). Nevertheless, this correlation was nonlinear and not easily extrapolated to the [1 µm–9 µm] aerosol (only 4.7%±0.6% of particles <0.5 µm but significant deposition in the TH deposition at 28%±17%). Additionally, particle deposition in the upper airways was mainly observed for particles with a median diameter of [1 µm–9 µm] (72%±17%). This experimental evidence was also supported by an *in vivo* study of pattern aerosol deposition [28].

Nevertheless, the airborne particles delivered by nebulizers could present high polydispersity, and thus the different mechanisms of deposition (impaction, sedimentation, diffusion) could occur simultaneously for a polydisperse aerosol. As an example, even if the AMAD of the atomizer nebulizer is 2.8 µm, significant amounts of FP and ultrafine particle fractions are also present (Figure 1, Table 2). The [1 µm–9 µm] aerosol has a GSD of 3.2, which is very broad, and it has many large particles that show high ET deposition. However, it could be a relevant particle size for environmental liquid droplets. The term “particulate matter” (also known as particle pollution) is a complex mixture including solid particles and liquid droplets found in air. These solid and liquid particles come in a wide range of sizes. However, it would be interesting to perform inhalation experiments with airborne particles within a narrower range of sizes (*i.e.,* low GSD). All things considered, some limitations can be underscored, such as possible errors in AMAD measurements because of liquid particle evaporation in transit to and through the ELPI impactor [29]. A cascade impactor can act as a warmer to evaporate the particles along the stages, decreasing the particle size and increasing the GSD.

### Impact of Airborne Particle Size on the Deposition Pattern in Terms of Inhaled Aerosol Fraction Deposited in the ET and TH Regions

The level of injury produced by inhaled toxicants depends on the dose received by the lungs and internal organs. Even if a dose expressed in toxicological studies in mg/kg of body mass or mg/cm^2^ of airway surface could not be calculated from these experiments, we provided an estimate of the percentage of inhaled material that deposits in the ET and TH regions of baboons at resting ventilation for three specific particle size distributions. In this sense, we calculated the emitted nebulizer fraction deposited in the ET and TH regions, knowing the aerosol amount deposited in each region of the respiratory tract and the emitted nebulizer fraction delivered by each nebulizer. We showed that the inhalation exposure to [0.25 µm–1 µm] or [0.15 µm–0.5 µm] radioactive aerosol led to the same inhaled fraction deposited in the TH region. However, the inhalation exposure to [1 µm–9 µm] or [0.25 µm–1 µm] radioactive aerosol led to the same inhaled fraction deposited in the ET region (Figure 3).

### Baboon–Human Comparison of *In Vivo* Experimental Pattern Deposition for the [1 µm–9 µm] Airborne Particles

Much information concerning inhalation toxicology has been collected from laboratory animals as human surrogates in aerosol inhalation studies. The comparative regional deposition in these laboratory animals can be considered helpful for interpreting, from a dosimetric viewpoint, the possible implications of animal toxicological results for humans. The *in vivo* experiments were conducted with baboons, which were used as a representative 2-year-old child respiratory tract model. Some studies have indicated that baboons are the most predictive primate species for extrapolating aerosol deposition data to humans [30]. Total inhaled aerosol deposition probability versus particle size is qualitatively similar for various mammals of similar body mass, despite airway anatomy differences, even if more species variation can be seen in regional particle deposition curves [31]. However, because anatomy and physiology of the respiratory system can significantly differ between species (specifically between baboons and children), several parameters must be taken into account to evaluate the usefulness of the baboon as an animal model of aerosol delivery in children. For example, it is necessary to compare the morphometry of baboon airways with the respiratory tract geometry of children. These correlations are critical for subsequent extrapolation of aerosol deposition findings in baboons to children. As an example, a large database was proposed for developing realistic age-dependent models of the human lung from infancy to young adulthood [32]. A morphometric comparison of the nasopharyngeal airway of a 3-year-old child and a 10-year-old baboon suggested a functional interspecific relationship between the nasal structure, cross-sectional area, and tracheobronchial region [15], [16]. According to the International Commission on Radiological Protection [33], a 1-year-old child has a tidal volume of 0.1 L and a minute ventilation of 3.7 L/min (approximately two-times more than that of the baboons in the study). We assumed the conclusion that the breathing parameters (such as the tidal volume) measured in the three studied baboons (Table 1) accorded quite well with those of a 1-year-old to 2-year-old child [34]. All things considered, the baboon appears to be a satisfactory *in vivo* respiratory model for a 2-year-old child. However, some limitations using baboons as child surrogates can also be underscored, such as the intra-animal anatomical variability or the effect of anesthesia on airway muscle tone, which, if airway geometry changed, would significantly affect the aerosol amount deposited.

Generally, the number of aerosol particles that reach the pulmonary region in children is lower than that in adults [6]. Because of a previous healthy volunteer scintigraphy study using the Atomiser NL11 nebulizer, a baboon–adult comparison of *in vivo* experimental pattern deposition for the [1 µm–9 µm] airborne particles can be proposed [35], [36]. A similar inhalation protocol was used to study the human and baboon deposition patterns, such as exposure route, delivery technique used (the same Atomisor NL11 nebulizer having the same AMAD and GSD), and definitions of specific respiratory tract regions. The human study included seven healthy men aged 21 to 36 years with a mean height of 181±3 cm and a mean weight of 77±10 kg who inhaled only via the nose [36].

The human study showed 73%±10% of aerosol deposited in airways was in the ET region and 27%±10% was in the TH region. Thus, we observed excellent correlation for the *in vivo* regional distribution in terms of total aerosol fraction deposited in human and baboon airways (ET region: 73%±10% in humans vs. 72%±17% in baboons; TH region: 27%±10% in humans vs. 28%±17% in baboons). This result led to the conclusion that differences in airway architecture or ventilation rate between adult and child respiratory models did not have a significant impact on the regional distribution of a [1 µm–9 µm] aerosol in terms of total aerosol fraction deposited in the airways with inhalation via the nasal route. This result is in good accordance with comparisons of deposition across different age groups performed in infants and in adults [11]. An alternative interpretation is that effects of differences in airway architecture and ventilation rate between adults and children were compensatory.

Nevertheless, the interspecies differences had a strong impact on the emitted nebulizer fraction that was deposited in the ET and TH regions. Based on the healthy volunteer scintigraphy study, if we calculate the emitted nebulizer fraction deposited (knowing the aerosol-emitted nebulizer fraction for the Atomiser NL11 nebulizer and the deposition fraction expressed as a percentage of the nebulizer charge), we could find an emitted nebulizer fraction of 25.2%±7% deposited in the ET region and an emitted nebulizer fraction of 8.3%±3.8% deposited in the TH region. Comparing these results to the baboon data (Table 3), we observed a six-fold higher value of emitted nebulizer fraction deposited in both the ET and TH regions in humans (ET region: 25.2%±7% in humans vs. 3.7%±1.6% in baboons; TH region: 8.3%±3.8% in humans vs. 1.3%±0.65% in baboons). We support the conclusion that this significant interspecies difference in terms of emitted nebulizer fraction deposited is attributable to differences in breathing parameters [37], [38]. We measured the volume of inspired air at 1.8±0.15 L/min in baboons (Table 1). Because it is commonly accepted that the volume of air that can be inspired is approximately 9 L/min in humans [33], we found that the volume of inspired air is five-fold higher in humans than in baboons (*i.e.*, 9 L vs. 1.8 L). Consequently, this result led us to think that the six-fold higher emitted nebulizer fraction deposited in the ET and TH regions in humans compared to baboons was mainly attributable to differences in inspired air volume. Thus, our results confirm that, for some sizes of airborne particles, regional deposition in humans and baboons is quite similar and appears to be species-independent. However, even if these different species are exposed to identical particles at an identical concentration, they will not receive the same particle mass per unit of exposure time because of their differences in tidal volume and breathing rate.

## Conclusion

Dose and persistence evaluations of inhaled aerosol particles are needed for children; data regarding the adult respiratory tract are also lacking, specifically for the submicron particle range. Knowledge of the regional distribution of inhaled submicron-size particles in the respiratory tract can help predict the sites of pathological changes attributable to airborne nanoparticle agglomerate exposure. Therefore, this study may add new data regarding this issue using the baboon animal model as a valuable strategy to assess such data.

The size-dependent regionalization of fine and ultrafine aerosol deposition *in vivo* within the baboon respiratory model provides an innovative aspect to this work, mainly because previous studies generally focused on determining globally or regionally deposited aerosol fractions in adult respiratory models. Results clearly demonstrated that aerosol deposition within the TH and ET regions varied substantially according to particle size. A high deposition in the ET region was observed for the [1 µm–9 µm] aerosol (72%±17%), whereas the [0.15 µm–0.5 µm] aerosol was almost exclusively associated with TH deposition (84%±4%). The [0.25 µm–1 µm] aerosol showed an intermediate deposition pattern, with 49%±8% of deposition in the ET region and 51%±8% of deposition in the TH region.

We established that a decrease of the intersubject variability and a targeted deposition in the TH region can be achieved by reducing the median activity diameter (AMAD) of airborne particles and by enhancing the amount of particles smaller than 0.5 µm. For toxicology issues, these results indicate the capacity of 200-nm to 300-nm airborne particles to preferentially access the lower airways, which possibly may be associated with a systemic translocation through the air–blood barrier of primary nanoparticles because these submicron-size structures should be de-agglomerated in body fluids. These data are of value not only for risk assessment but also for inhalation therapy strategy evaluation and dosing.

## Supporting Information

Checklist S1 The ARRIVE (Animal Research: Reporting *In Vivo* Experiments) guidelines.
(PDF)
